# CRISPR/Cas9 Genome Editing of Epidermal Growth Factor Receptor Sufficiently Abolished Oncogenicity in Anaplastic Thyroid Cancer

**DOI:** 10.1155/2018/3835783

**Published:** 2018-04-12

**Authors:** Li-Chi Huang, Ka-Wai Tam, Wei-Ni Liu, Chun-Yu Lin, Kai-Wen Hsu, Wen-Shyang Hsieh, Wei-Ming Chi, Ai-Wei Lee, Jinn-Moon Yang, Ching-Ling Lin, Chia-Hwa Lee

**Affiliations:** ^1^Department of Endocrinology and Metabolism, Cathay General Hospital, Taipei, Taiwan; ^2^Department of Internal Medicine, School of Medicine, College of Medicine, Taipei Medical University, Taipei, Taiwan; ^3^Division of General Surgery, Department of Surgery, Shuang Ho Hospital, Taipei Medical University, New Taipei City, Taiwan; ^4^Department of Surgery, School of Medicine, College of Medicine, Taipei Medical University, Taipei, Taiwan; ^5^Graduate Institute of Medical Science, College of Medicine, Taipei Medical University, Taipei, Taiwan; ^6^Institute of Bioinformatics and Systems Biology, National Chiao Tung University, Hsinchu, Taiwan; ^7^Research Center for Tumor Medical Science, China Medical University, Taichung, Taiwan; ^8^Department of Laboratory Medicine, Shuang Ho Hospital, Taipei Medical University, Taipei, Taiwan; ^9^Department of Anatomy and Cell Biology, School of Medicine, College of Medicine, Taipei Medical University, Taipei, Taiwan; ^10^Department of Biological Science and Technology, National Chiao Tung University, Hsinchu, Taiwan; ^11^School of Medical Laboratory Science and Biotechnology, College of Medical Science and Technology, Taipei Medical University, Taipei, Taiwan; ^12^Ph.D. Program in Medicine Biotechnology, College of Medicine, Taipei Medical University, Taipei, Taiwan; ^13^Comprehensive Cancer Center of Taipei Medical University, Taipei, Taiwan

## Abstract

Anaplastic carcinoma of the thyroid (ATC), also called undifferentiated thyroid cancer, is the least common but most aggressive and deadly thyroid gland malignancy of all thyroid cancers. The aim of this study is to explore essential biomarker and use CRISPR/Cas9 with lentivirus delivery to establish a gene-target therapeutic platform in ATC cells. At the beginning, the gene expression datasets from 1036 cancers from CCLE and 8215 tumors from TCGA were collected and analyzed, showing EGFR is predominantly overexpressed in thyroid cancers than other type of cancers (*P* = 0.017 in CCLE and *P* = 0.001 in TCGA). Using CRISPR/Cas9 genomic edit system, ATC cells with EGFR sgRNA lentivirus transfection obtained great disruptions on gene and protein expression, resulting in cell cycle arrest, cell growth inhibition, and most importantly metastasis turn-off ability. In addition, the FDA-approved TKI of afatinib for EGFR targeting also illustrates great anticancer activity on cancer cell death occurrence, cell growth inhibition, and cell cycle arrest in SW579 cells, an EGFR expressing human ATC cell line. Furthermore, off-target effect of using EGFR sgRNAs was measured and found no genomic editing can be detected in off-target candidate gene. To conclude, this study provides potential ATC therapeutic strategies for current and future clinical needs, which may be possible in increasing the survival rate of ATC patients by translational medicine.

## 1. Introduction

Anaplastic carcinoma of the thyroid (ATC), also called undifferentiated thyroid cancer, is the least common and most aggressive and deadly thyroid gland malignancy of all thyroid cancers. Patients are usually in their 60s–70s at presentation, having an average median survival of five months, and most patients with ATC do not live one year from the day they are diagnosed [1, 2]. Due to the extremely aggressive behavior of ATC, the American Joint Committee on Cancer (AJCC) defines all of its stages as stage IV [3]. Currently, the cause of ATC still remains unknown and there are not any known links between ATC and any behavioral factors or lifestyle factors. As far as we know, ATC does not respond to radioactive iodine (^131^I) therapy or thyroid-stimulating hormone (TSH) suppression with levothyroxine used. In other words, no effective therapeutic options were available for patients with ATC resistant to radioiodine. Even the conventional therapies such as external beam radiotherapy and chemotherapy are not able to prolong survival either as a single therapeutic agent or as combination therapy strategy [4].

Until recently, several of the tyrosine kinase inhibitors (TKIs) have been evaluated in advanced thyroid cancers for their ability to block tyrosine kinase receptors and/or other kinases involved in cell proliferation and tumoral transformation of the thyroid cells. In addition to that, the American Thyroid Association Guideline suggests the combination of surgery, radioactive iodine ablation, and chemotherapy may improve outcomes in ATC. Because of the high aggressiveness of ATC, previous study has suggested that vascular endothelial growth factor receptor (VEGFR), platelet-derived growth factor receptor (PDGFR), fibroblast growth factor receptor (FGFR), and hepatocyte growth factor receptor (HGFR/c-Met) in ATC are associated with clinical features of the disease [5]. Multikinase TKIs of lenvatinib, cabozantinib, vandetanib, and sorafenib that target VEGFR, PDGFR, FGFR, KIT, and RET pathways are the four target therapy drugs approved by the Food and Drug Administration (FDA) for advanced thyroid cancer [6]. Despite the successful TKI clinical trials in thyroid cancer, many aspects of uncertainty in the treatment of thyroid cancer patients with targeted therapies remain to be elucidated. For instance, since TKI treatment has recently indicated on thyroid cancers, it remains unknown if the use of TKIs will prolong the life of thyroid cancer patients. It is also not certain that the limitations of TKIs such as the escape phenomenon and the restarting growth of some lesions or the appearance of new cancer will occur in the thyroid. Thus, we need to discover new biomarkers and develop new type of therapeutic forms for ATC patients in the future.

Several gene-edit technologies, such as zinc finger nucleases (ZFNs) and transcription activator-like effector nucleases (TALENs), have been utilized for therapeutic application in animal and cellular cancer models [7–9]. Until recently, a new clustered regularly interspaced short palindromic repeats (CRISPR)/Cas9 system started a new era of genome editing [10–12]. CRISPR/Cas9 generates double-strand breaks (DSBs) at target sites by recognizing 20- or 24-nt sequences that match an engineered gRNA and a protospacer adjacent motif (PAM) located downstream of the target sequence. The subsequent cellular DNA repair process introduces the desired insertions, deletions, or substitutions at the target site [13]. The potential application of the preclinical CRISPR/Cas9 therapeutic strategies against human cancers and furthermore to overcome the limitations in translating therapeutic CRISPR/Cas9 into clinical use is highly expected.

In our previous study, we found that *α*9-nAChR plays an important role in manipulating breast cancer metastasis ability in the “Triple-Negative Breast Cancers Spontaneous Pulmonary Metastasis Mouse Model” developed by our research team [14]. Using customized sgRNAs for CRISPR/Cas9 gene editing on *α*9-nAChR DNA locus of MDA-MB-231 cells through lentivirus infection, we found a significant *α*9-nAChR gene sequence mixture around the expected Cas9 cleavage point using Sanger DNA sequencing and Tracking of Indels by Decomposition (TIDE) analysis. Further experimental confirmations of *α*9-nAChR gene-editing efficiency were also carried out by applying RNA-guided engineered nuclease-restriction fragment length polymorphism (RGEN-RFLP) assay and α9-nAChR protein measurement. Using similar parameters, in this current study, we aim to discover novel biomarker of ATC by analyzing cancer cell and tumor gene expression datasets and use currently available TKI against it for clinical ATC therapy. This new study utilizes the CRISPR/Cas9 gene-editing strategy to eliminate the identified protein and its oncogenic activity in vitro. Through this combination, we are able to generate a gene-based target therapy, providing both short-term and long-term translational information for the needs of current clinic and future drug development on ATC treatment.

## 2. Material and Methods

### 2.1. Cell Lines and Culture Conditions

Human mammary gland epithelial adenocarcinoma cell line MDA-MB-231, human gland epithelial squamous cell carcinoma cell line SW579 (anaplastic thyroid carcinoma) [15, 16], and human kidney epithelial Phoenix-ECO cells were purchased from the American Tissue Culture Collection (ATCC, Manassas, VA) and maintained in Dulbecco's Modified Eagle Medium: Nutrient Mixture F-12 (DMEM/F-12) Media (Gibco, USA). The cells were incubated with 10% (*v*/*v*) fetal bovine serum (FBS, Biological Industries, Israel). The supplement of 100 units/ml penicillin and 100 mg/ml streptomycin was used and cultured in a 37°C incubator with 5.0% CO_2_. The medium was replaced every two days, and when cells reached 80% confluence, they were passaged using 0.25% trypsin/EDTA (Gibco, USA).

### 2.2. Design of On-Target and Off-Target sgRNAs for the *ABL* Gene

We followed the methods of Huang et al. 2017 [14]. Custom sgRNAs for *EGFR* were designed using the MIT CRISPR Design website (http://crispr.mit.edu) with the sequence of *EGFR* (NM_005228). This website provides both on-target sequences and off-target possibilities. We selected the highest scoring off-target sequences in the *EGFR* protein-coding region sgRNA_2 (mismatch = 3), whereas no potential off-target candidate gene (mismatch < 4) can be found in sgRNA_1.

### 2.3. Flow Cytometry Analysis

The MDA-MB-231 and SW579 cells of lentivirus infected or afatinib treated were collected and stored in a 75% alcohol-PBS solution for flow cytometry analysis. The forward scatter (FSC) measurement was conducted by FACSCalibur (BD Biosciences).

### 2.4. Real-Time Quantitative Polymerase Chain Reaction (Q-PCR)

The human metastatic-related gene primers are listed in the Supplementary Table available here. All oligo primers were synthesised by Genomics BioSci and Tech (Taipei, Taiwan). A LightCycler thermocycler (Roche Molecular Biochemicals, Mannheim, Germany) was used for Q-PCR analysis. One microliter of the sample and master mix was first denatured for 10 minutes at 95°C and then incubated during 40 cycles (denaturation at 95°C for 5 seconds, annealing at 60°C for 5 seconds, and elongation at 72°C for 10 seconds) to detect fluorescent intensity. All of the PCR samples underwent melting curve analysis for nonspecific PCR product detection. The gene expression results from the Q-PCR analysis were normalized with human *β*-glucuronidase (GUS) expression as an internal control using the built-in Roche LightCycler Software, Version 4.

### 2.5. Absolute Q-PCR

To generate the absolute quantitative standard curve for Q-PCR analysis on lentivirus copy number measurement, we used the PCR product of the mouse GUS gene and cloned it with the TA cloning vector (pTA® Easy Cloning Kit), which was purchased from Genomics BioSci and Tech (Taipei, Taiwan). After following the steps of gene sequencing, *E*. *coli* amplification, plasmid purification, and determination of molecular weight, the copied GUS genes were calculated and diluted by 10^8^ to 10^2^ per *μ*l. Each copied gene was measured for accuracy and liner correlation.

### 2.6. Protein Extraction, Western Blotting, and Antibodies

For western blot analysis, the SW579 cells of either lentivirus infected or afatinib treated were washed once with ice-cold PBS and lysed with radioimmunoprecipitation assay (RIPA) lysis buffer containing protease inhibitors. Fifty micrograms of protein from each sample was resolved by sodium dodecyl sulfate polyacrylamide gel electrophoresis (SDS-PAGE) and transferred to a nitrocellulose membrane. The anti-P53 (SC-5243), anti-p-ERK (SC-7383), and anti-GAPDH (SC-32233) antibodies were purchased from Santa Cruz Biotechnology (Santa Cruz, CA, USA); the anti-P21 (GTX629543) and anti-p-AKT (GTX128414) were purchased from GeneTex Inc. (Irvine, CA, USA); the anti-PARP (AFFN-PARP1-17B10-s) antibody was purchased from the Developmental Studies Hybridoma Bank (Iowa City, IA, USA); and the anti-EGFR (#4267) antibody was purchased from Cell Signaling Technology (Danvers, MA, USA). The secondary antimouse and rabbit antibodies were purchased from Santa Cruz Biotechnology. All of the primary antibodies were used at 1000 dilution and overnight hybridization, followed by a one-hour incubation with a 1 : 4000 dilution of the secondary antibodies.

### 2.7. Lentiviral Production and Cell Transduction

Lentiviral particles were produced by the transient transfection of Phoenix-ECO cells (CRL-3214) using TransIT®-LT1 Reagent **(**Mirus Bio LLC, Madison, WI, USA). Guide oligonucleotides were phosphorylated, annealed, and cloned into the BsmBI site of the lentiCRISPR v2 vector (Addgene, 52961, kindly provided by Feng Zhang) according to the Zhang laboratory protocol [11] (F. Zhang lab, MIT, Cambridge, MA, USA). The lentiCRISPR construct or the pLJM1-EGFP plasmid (Addgene plasmid #19319, a gift from David Sabatini) was cotransfected with pMD2.G (Addgene plasmid #12259) and psPAX2 (Addgene plasmid #12260, both kindly provided by Didier Trono, EPFL, Lausanne, Switzerland). Lentiviral particles were collected at 36 and 72 hours before being concentrated with Lenti-X Concentrator® (Clontech, Mountain View, CA., USA). The lentivirus concentration for each gene was quantified by Q-PCR. SW579 cells were infected in a 6 cm dish, with each well containing 2.5 × 10^5^ cells and different concentrations of lentivirus. Two days after transfection, the medium was replaced with 4 mg/ml puromycin for two days of cell selection. Lentivirus-transfected cells were recovered two days before the experiments.

### 2.8. Sequencing of Single-Guide RNA (sgRNA) Target Sites

Genomic DNA was extracted, and PCR amplified the EGFR gene region using the exon 3 or exon 9 DNA primers which are listed in the Supplementary Table. The PCR products were purified using a PCR Clean-up Purification Kit and were sequenced by the Sanger method using forward PCR primers. The editing efficiency of the sgRNAs and the potential induced mutations were assessed using Tracking of Indels by Decomposition (TIDE) software (https://tide-calculator.nki.nl; Netherlands Cancer Institute), which only required two Sanger sequencing runs from wild-type cells and mutated cells.

### 2.9. RNA-Guided Engineered Nuclease-Restriction Fragment Length Polymorphism (RGEN-RFLP) Assay

PCR products (approximately 100 ng per assay) were incubated for 30 minutes at 37°C with the Cas9 protein (New England Biolabs (NEB), Beverly, MA, USA) and *α*9-nAChR sgRNA in 10 *μ*l of NEB buffer 3. After cleavage, RNase A (2 *μ*g) was added, and the reaction mixture was incubated for 15 minutes at 37°C to remove RNA. Next, proteinase K (2 *μ*g) was added, and the reaction mixture was incubated for 15 minutes at 58°C to remove the Cas9 protein. The products were resolved on 2% agarose gels and visualized with ethidium bromide (EtBr) staining.

### 2.10. Gene Expression Datasets

The Cancer Cell Line Encyclopedia (CCLE- https://cancergenome.nih.gov/) and The Cancer Genome Atlas (TCGA- https://cancergenome.nih.gov/) gene microarray datasets were collect and analyzed by EGFR member expressions.

### 2.11. Immunohistochemistry

Immunocytochemistry of EGFR protein expressions in human papillary (antibody ID HPA018530, patient ID 2623), follicular (antibody ID CAB000035, patient ID 923), anaplastic (antibody ID CAB000035, patient ID 306) thyroid cancers, and normal thyroid gland tissues (antibody ID CAB068186, patient ID 1922) were obtained from Human Protein Atlas (http://www.proteinatlas.org).

### 2.12. Statistical Methods

All data were expressed as mean ± SD, and Student's *t*-test analysis was performed for the pairwise samples. All statistical comparisons were performed using SigmaPlot graphing software (San Jose, CA, USA) and Statistical Package for the Social Sciences v.13 (SPSS, Chicago, IL, USA). A *P* value < 0.05 was considered statistically significant, and all statistical tests were two-sided.

## 3. Results

### 3.1. Characteristics of the EGFR Family Expressions in Thyroid Cancer Cells

**Figure 1:**
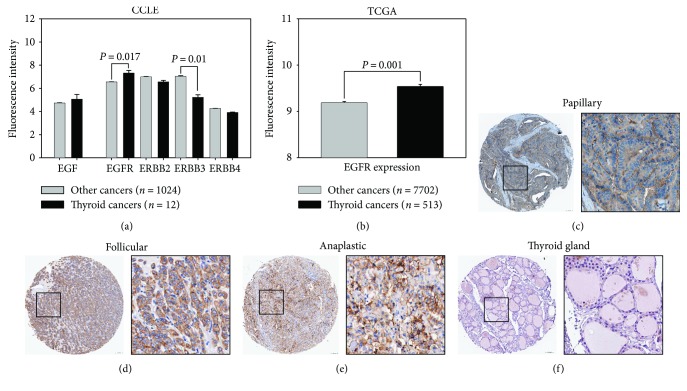
Characteristics and analysis of the EGFR family expressions in thyroid cancer cells: (a) determination of EGFR member expressions using CCLE microarray gene database of all 1036 cancer cells. The expressions are divided into thyroid cancer cells (12 cells) and other cancer cell types (1024 cancer cells). (b) Determination of EGFR expression using TCGA clinical tumor microarray gene database of all 8215 tumors. The gene expressions are divided into thyroid tumors (513 tumors) and other tumor types (7702 tumors). The gene expressions were determined by fluorescence intensity. The statistical significance is given between groups by Student's *t*-test. Immunocytochemistry of EGFR protein expressions (brown stain) in (c) human papillary, (d) follicular, (e) anaplastic thyroid cancers, and (f) normal thyroid gland tissues was obtained from the Human Protein Atlas and presented in low- (left photo) and high-power fields (right photo). Scale bar = 50 *μ*M.

The CCLE and other microarray database have made great effort to explore the genomic landscape of cancers [17]. In order to investigate the expression of EGFR family members in cancers, the gene expression profiles of 1036 cancer cells from CCLE database are classified into 24 tumor types (Figure 1(a)). We found that, as compared to the other 1024 cancer cells, the overall EGFR expression in 12 thyroid cancer cells is significantly overexpressed with 1.7-folds (thyroid cancers versus other cancers = 7.302 ± 0.24 versus 6.53 ± 0.03 fluorescence intensity-log^2^, *P* = 0.017), whereas the overall expressions of ERBB2, ERBB4, and EGF show similar expressions in all cancer cells. Interestingly, we found the overall ERBB3 expression in thyroid cancers is significantly underexpressed in other cancer types with 3.51-folds (thyroid cancers versus other cancers = 5.21 ± 0.24 versus 7.02 ± 0.08 fluorescence intensity-log^2^, *P* = 0.01), indicating ligand binding to this “kinase-dead” ERBB3 receptor leads to dimerization with another kinase-competent family member which is not acquired in thyroid cancer development [18, 19]. In addition, ERBB4 is the least expressed EGFR member in all cancer cell lines, even though some studies have implied that ERBB4 plays an important role in cancer development [20, 21]. The significance of EGFR expression is also evaluated in human thyroid cancers using TCGA clinical cancer database of all 8215 tumors (Figure 1(b)). Similarly, we found that, as compared to the other type of 7702 tumor tissues, the overall EGFR expression in 513 thyroid cancers is significantly overexpressed with 1.35-folds (thyroid cancers versus other cancers = 9.535 ± 0.049 versus 9.1838 ± 0.037 fluorescence intensity-log^2^, *P* = 0.001). To confirm this finding, EGFR expressions in both normal thyroid gland and thyroid cancer tissues were obtained from the Human Protein Atlas [22]. In tissue microarray sections, it is shown that EGFR is highly expressed in papillary (Figure 1(c)), follicular (Figure 1(d)), and the deadliest anaplastic (Figure 1(e)) subtype of thyroid cancers, compared to the low EGFR expression found in the normal thyroid gland (Figure 1(f)). These evidence indicates that EGFR has great potential to use as a biomarker and target for thyroid cancer therapy.

### 3.2. *EGFR* Gene Targeting Using the CRISPR/CAS9 System

**Figure 2:**
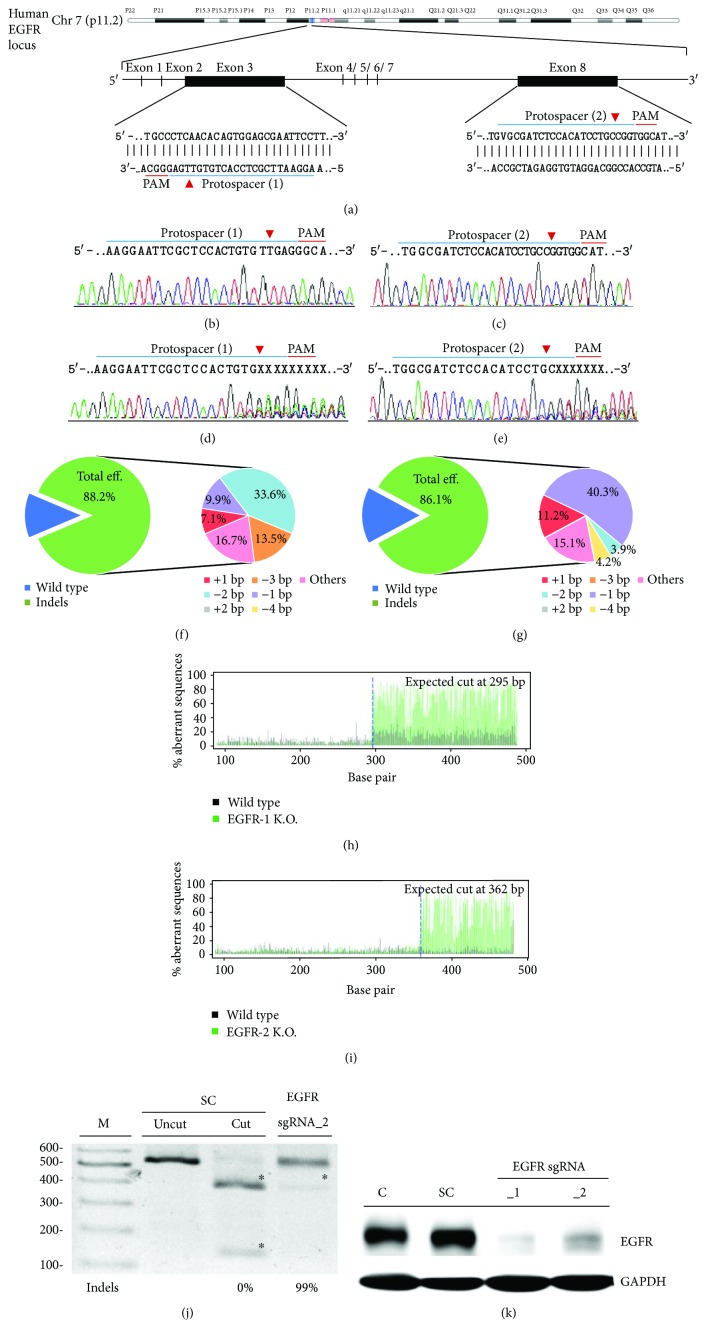
*EGFR* gene targeting in MDA-MB-231 cells using the CRISPR/CAS9 system: (a) schematic representation of the human *EGFR* DNA locus and two protospacer sequences (blue underline) for editing. The arrowhead indicates the expected Cas9 cleavage site. The protospacer adjacent motif (PAM, red underline) is the motif required for Cas9 nuclease activity. Scrambled (SC) sgRNA and *EGFR* sgRNA were delivered to MDA-MB-231 cells by lentivirus. After transduction, DNA from virus-infected cells was purified and subjected to Sanger sequencing of *EGFR* exon 3 and exon 9. (b, c) Wild-type *EGFR* sequences in MDA-MB-231 cells. (d) *EGFR* sgRNA_1 and (e) *EGFR* sgRNA_2 producing a mixture of sequences around the expected Cas9 cleavage point in a pool of gene-edited cells after lentivirus transduction. TIDE algorithm analysis of the *EGFR* gene-edited sequence (indels, insertions, and deletions) showed a high editing efficiency in MDA-MB-231 cells. The pie charts show the percentages of indels in the *EGFR* gene edited by (f) *EGFR* sgRNA_1 and (g) *EGFR* sgRNA_2. The gene-editing efficiency of the two sgRNAs is presented in green, while the two most common −1 and −2 indels are presented in purple and blue, respectively. The original TIDE algorithm analysis is shown for (h) *EGFR* sgRNA_1 and (i) *EGFR* sgRNA_2 virus transfected on MDA-MB-231 cells, compared to SC MDA-MB-231 cells. The panels illustrate the aberrant sequence signal in the scrambled (green versus black). (j) The *EGFR* gene in MDA-MB-231 cells analyzed with the RGEN-RFLP assay to measure the gene-editing efficiency. The agarose image of *EGFR* gene cleavage with specific sgRNA and Cas9 addition represents the indel percentage in the gene-editing pool. The fragments of cleavage DNA were highlighted with an asterisk. (k) Western blot analysis of EGFR protein expression. (c) Image showing parental and SC sgRNA control MDA-MB-231 cells; EGFR expression was significantly reduced in *EGFR* sgRNA_1- and *EGFR* sgRNA_2-transduced MDA-MB-231 cells.

Next, we investigated the utility of CRISPR/Cas9 genome editing by targeting two custom-designed protospacers on the *EGFR* locus on chromosome 7 with lentivirus delivery system using the MIT CRISPR Design website (http://crispr.mit.edu) with the sequence of *EGFR* (NM_005228.3). According to our previous study, MDA-MB-231 is a lentivirus easy-transfected cell. In the pilot study, we used MDA-MB-231 as a target cell to investigate lentivirus-mediated EGFR gene-edit efficiency. As shown in the *EGFR* genomic map (Figure 2(a)), protospacer 1 targets the negative strand of exon 3 *EGFR* gene, whereas protospacer 2 targets the plus strand of exon 9 *EGFR* gene. Transduction of MDA-MB-231 cells with the target scrambled (SC) lentivirus produced a wild-type *EGFR* sequence, as assessed by Sanger sequencing (Figures 2(b) and 2(c)), with no evidence of gene editing. However, transduction with the *EGFR* sgRNA_1 lentivirus, which carries protospacer 1 (Figure 2(d)), or the *EGFR* sgRNA_2 lentivirus, which carries protospacer 2 (Figure 2(e)), led to multiple gene disruptions at the predicted cleavage sites (red arrowhead) of both protospacers. In addition, lentivirus infection of both *EGFR* sgRNA_1 (Figure 2(f)) and *EGFR* sgRNA_2 (Figure 2(g)) was shown by TIDE analysis to have considerable gene-editing efficiency: 88.2% and 86.1% of the cell pool was edited, respectively. The most frequent mutations in the *EGFR* sgRNA_1 cell pool were 2 bp deletion (33.6%) and other mutations (13.5%), whereas the frequently predicted mutations in the *EGFR* sgRNA_2 cell pool were 1 bp deletion (40.3%) and other mutations (11.2%). Both *EGFR* sgRNAs caused significant gene disruptions in the targeted regions, with mutations primarily at the predicted cleavage sites (Figures 2(h) and 2(i)). Next, we performed RGEN-RFLP analysis to quantify the *EGFR* gene-editing efficiency. RGEN-RFLP analysis of the gene-editing efficiency on *EGFR* DNA region showed that the SC sgRNA without Cas9 did not cleave DNA (uncut, Figure 2(j)); however, the SC sgRNA with Cas9 fully cleaved DNA into fragments (cut, with an asterisk), indicating a 100% wild-type DNA sequence without gene disruption. As an example, the DNA load of *EGFR* sgRNA_2-transfected MDA-MB-231 cells showed a 99% gene edit, indicating that lentivirus-delivered *EGFR* sgRNA_2 had a great gene-editing efficiency on MDA-MB-231 cells. EGFR protein expression was also assessed by western blot (Figure 2(k)). As expected, MDA-MB-231 cells infected with the SC sgRNA virus expressed strong EGFR protein, whereas infection with the *EGFR* sgRNA_2 virus, EGFR protein levels were significantly decreased.

### 3.3. Optimization of *EGFR* Gene Targeting Viral Transduction Conditions in SW579 Cells

**Figure 3:**
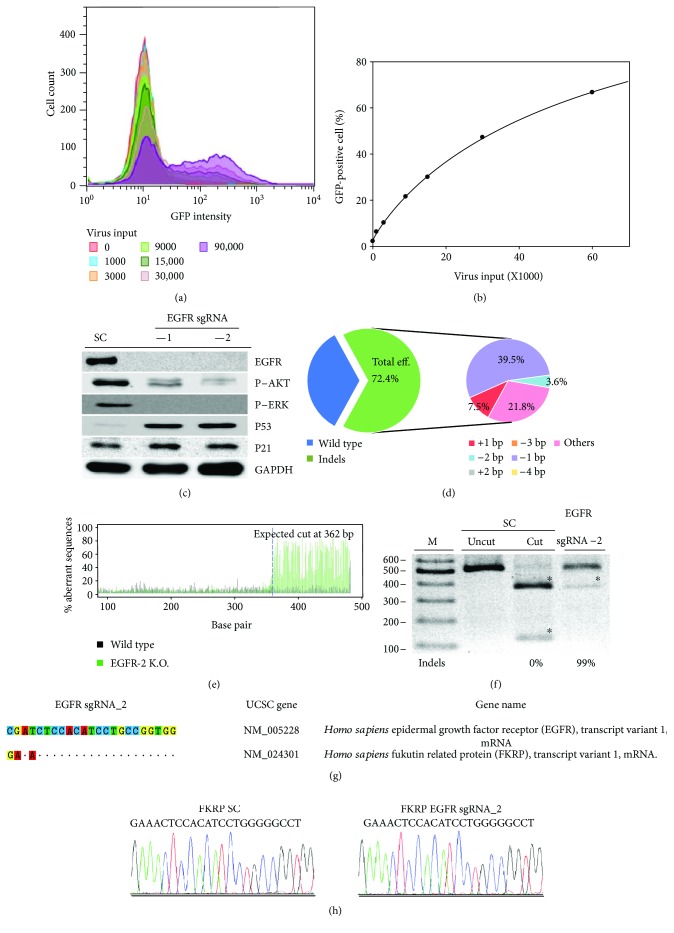
Optimization of viral transduction conditions for human SW579 cells: (a) the purified and concentrated PLJM1-GFP lentivirus was measured as virus copy number by Q-PCR analysis. SW579 cells were seeded in a 6 cm dish and infected with 1000-, 3000-, 9000-, 15,000-, 30,000-, and 90,000-fold concentrations of virus to SW579 cell number for three days. The GFP-positive (infected) cell population was assessed using flow cytometry. (b) The image shows the linear curve comparison of virus input and the GFP-positive cell population. (c) The image shows the western blot analysis of EGFR protein expression on SC sgRNA control SW579 cells; EGFR levels were significantly reduced in *EGFR* sgRNA_1- and *EGFR* sgRNA_2-transduced SW579 cells. The downstream signaling proteins such as phosphor-AKT and phosphor-ERK were also analyzed in *EGFR* sgRNA_1- and *EGFR* sgRNA_2-transduced SW579 cells. The tumor-suppressive proteins such as P53 and P21 were found to be induced in EGFR gene-edited cells. GAPDH served as internal control. (d) TIDE algorithm analysis of the *EGFR* gene-edited sequence (indels, insertions, and deletions) showed a high editing efficiency in SW579 cells. The pie charts show the percentages of indels in the *EGFR* gene edited by *EGFR* sgRNA_2. The gene-editing efficiency of the two sgRNAs is presented in green, while the two most common −1 and other mutations are presented in purple and pink, respectively. (e) The panels illustrate the aberrant sequence signal in the scrambled (green versus black). (f) The *EGFR* gene in SW579 cells was analyzed with the RGEN-RFLP assay to measure the gene-editing efficiency. The agarose image of *EGFR* gene cleavage with specific *EGFR* sgRNA_2 and Cas9 addition represents the indel percentage in the gene-editing pool. The fragments of cleavage DNA were highlighted with an asterisk. (g) The CRISPR Design website was used to predict off-target candidate genes for both the *EGFR* sgRNA_1 and *EGFR* sgRNA_2 viruses. To note, no potential off-target candidate gene was predicted for *EGFR* sgRNA_1 (mismatch < 4). Similarities are presented as dots, and mismatch sites are indicated by nucleotide substitution. (h) Sanger sequencing of SW579 cells infected with SC and *EGFR* sgRNA_2 virus was used to examine potential indels in off-target candidate genes.

Several cell types are considered difficult-to-transfect cells, including the primary human fibroblasts, human umbilical vein endothelial cells, Jurkat cells, and leukemia cells [23]. In order to optimize the cell transduction efficiency of SW579 cells, an EGFR-expressing human ATC cell line [24], we transduced SW579 cells with different concentrations of the pLJM1-EGFP lentivirus (from 1000-fold to 90,000-fold). After three days, the number of GFP-positive SW579 cells was determined by flow cytometry (Supplementary Figure 1); this number was gradually increased after infection with increasing concentration of virus and reached maximum after infection with the 90,000-fold concentration (purple, Figure 3(a)). We next compared the number of GFP-positive SW579 cells and the lentivirus input; the linear curve showed that the 90,000-fold virus concentration reached 69% GFP-positive of the cell population (Figure 3(b)), whereas additional virus may not significantly increase the transduction efficiency in SW579 cells. In the following experiments, we used a 90,000-fold virus concentration for the following *EGFR* sgRNA delivery on SW579 cells. To understand the protein redundancy by *EGFR* sgRNA editing, EGFR protein expression was assessed by western blot, showing a significant decrease with the *EGFR* sgRNA_1 and sgRNA_2 virus-infected SW579 cells, compared to SC lentivirus-infected cells (Figure 3(c)). The cell growth-signaling proteins, such as AKT and ERK activation (phosphorylation form), were dramatically inhibited in *EGFR* gene-edited SW579 cells; such result also induced the expression of tumor-suppressive proteins, such as P53 and P21. After finding the great impacts of cell survival and signal transduction by the lentivirus-mediated *EGFR* gene editing, we investigated the utility of CRISPR/Cas9 genome editing in SW579 cells. The gene-edit efficiency by TIDE analysis on *EGFR* sgRNA_2 lentivirus infection shows to have considerable efficiency with 72.4% of the cell pool that was edited (Figure 3(d)). The most frequent mutations in the *EGFR* sgRNA_2 cell pool were 1 bp deletion (39.5%) and other mutation (21.8%). The *EGFR* sgRNA_2 caused gene disruptions in the targeted regions, with mutations primarily at the predicted cleavage sites (Figure 3(e)). RGEN-RFLP analysis of the gene-editing efficiency of the *EGFR* DNA region on SW579 cells showed that the SC sgRNA without Cas9 did not cleave DNA (uncut, Figure 3(f)); however, the SC sgRNA with Cas9 fully cleaved DNA into fragments (cut, with an asterisk), indicating a 100% wild-type DNA sequence without gene disruption. The DNA load of *EGFR* sgRNA_2-transfected SW579 cells showed a 99% gene edit, indicating that lentivirus-delivered *EGFR* sgRNA_2 had a great gene-editing efficiency on SW579 cells. Here, we used several techniques, including Sanger DNA sequencing, TIDE, RGEN-RFLP, and western blot, to determine which *EGFR* sgRNA had greater gene-editing efficiency and protein translation inhibition. The results from this study indicate that even in difficult-to-transfect cells, a high level of gene editing is possible with a lentivirus and an optimized sgRNA, suggesting the future potential for gene therapy in vitro and in vivo (patients). It is critical to determine whether the CRISPR/Cas9 technology causes unexpected cleavage events at similar DNA sequences or under other circumstances. We chose those with highly similar sequences to the *EGFR* sgRNA_2 as candidates for off-target cleavage (mismatch = 3), whereas no potential off-target candidate gene can be found in sgRNA_1 (mismatch < 4), and evaluated the genes by Sanger sequencing. The gene with a highly similar DNA sequence to the *EGFR* sgRNA_2 sequence is a fukutin-related protein (FKRP, Figure 3(g)), showing no genomic editing occurring in both SC and EGFR sgRNA_2 virus-infected SW579 cells (Figure 3(h)). This result validates the great specificity of the CRISPR/Cas9 system in targeting the *EGFR* gene on ATC cells, indicating the biosafety of using genomic editing in the future clinic.

### 3.4. The EGFR Abolished SW579 Cells Inhibiting Cancer Cell Growth and Metastasis Ability

**Figure 4:**
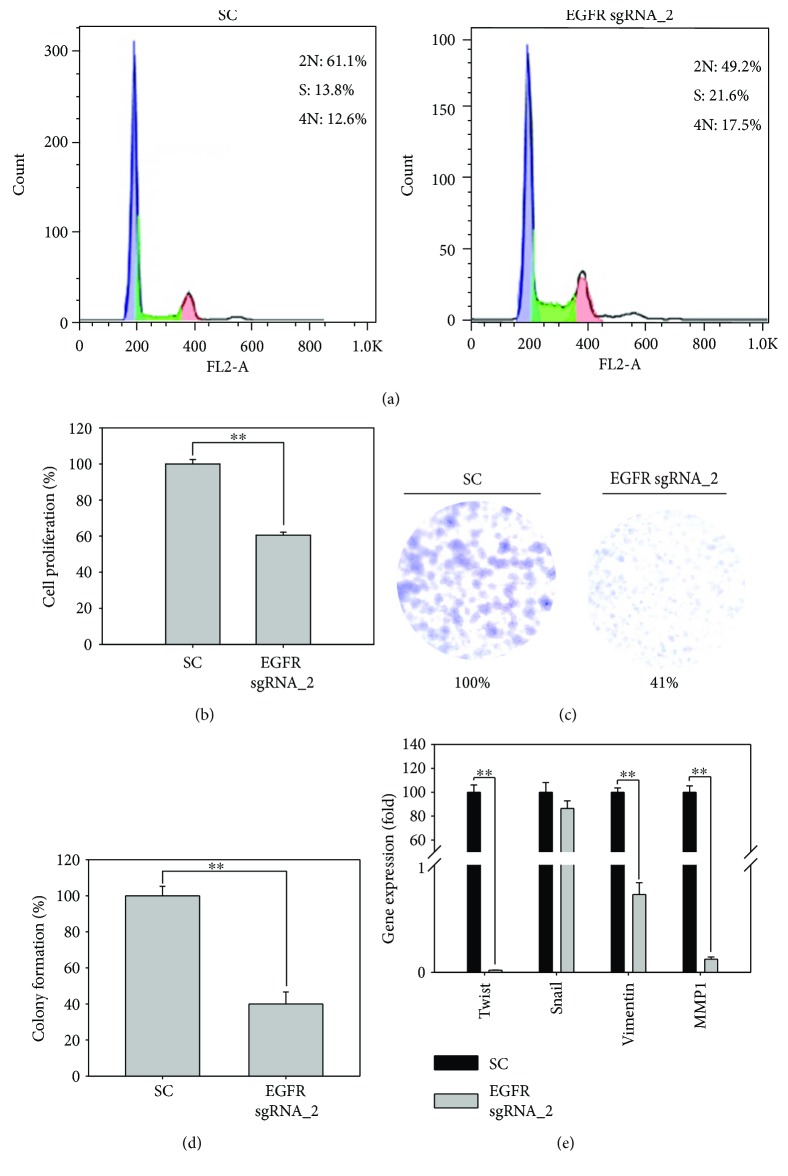
Functional assays of *EGFR* gene-edited SW579 cells: (a) The image shows the cell cycle distribution of SC sgRNA- and *EGFR* sgRNA_2-infected SW579 cells by flow cytometry analysis. (b) SW579 cell proliferation of SC sgRNA- and *EGFR* sgRNA_2-infected SW579 cells was observed for 4 days and determined by MTT assay. (c) Colony formation of SC sgRNA- and *EGFR* sgRNA_2-infected SW579 cells was measured by 0.5% crystal violet staining. (d) The cell colony numbers for both SC sgRNA- and *EGFR* sgRNA_2-infected SW579 cells were counted by ImageJ and presented in the percentage of SC control. (e) Q-PCR analysis on metastasis-related gene expressions, such as TWIST, SNAIL, VIMEMTIN, and MMP1, on SC sgRNA- and *EGFR* sgRNA_2-infected SW579 cells. All the gene expressions were normalized by their *GUS* gene level and presented in the percentage of SC. The statistical significance between groups is compared by Student's *t*-test. The *P* value less than 0.01 is presented with two asterisks.

To investigate the biofunction of EGFR in anaplastic thyroid cancers, we analyzed the cell population in each cell cycle phase on *EGFR* sgRNA_2-transfected SW579 cells. The cell cycle distribution assay demonstrated that EGFR abolished and largely enhanced the cell population at S phase from 13.8% of SC to 21.6% of *EGFR* sgRNA_2 virus transfection (Figure 4(a)). Next, we want to know whether this S phase latency of the cell cycle impacts the cell growth; MTT assay was used to determine the cell growth with or without EGFR editing. The data clearly shows the cells with *EGFR* sgRNA_2 virus transfection significantly reduced the cell growth by 40%, compared to SC virus transfection by a four-day observation (*P* = 0.0001, Figure 4(b)). With a long-term cell growth assay (Figure 4(c)), colony formation shows *EGFR* sgRNA_2 virus-transfected SW579 cells obtained a dramatic loss of colony formation ability than SC virus-transfected cells, with 41% of *EGFR* sgRNA_2 and VS 100% of SC (*P* = 0.001, Figure 4(d)), respectively. In addition, anaplastic thyroid cancer is infamous of the deadliest cancer in human; most of the patients are dead in six months after diagnosis and with less than 2% can survive more than five years. Thus, we evaluated whether this EGFR genomic edit system inhibits metastatic genes in SW579 cells. In Figure 4(e), *EGFR* sgRNA_2 virus-transfected cells almost completely abolished gene expressions of *TWIST*, *VIEMENTIN*, and *MMP1*, while the metastasis transcription factor *SNAIL* level remained unchanged, compared to SC cells. All these evidences strongly support the great potential of using *EGFR* CRISPR/Cas9 system to inhibit the malignancy in anaplastic cancer cells. It is worth to note that the highly efficient transfection effect of lentivirus as a carrier for gene knockout is the most approved strategy for gene therapy, suggesting a promising strategy for gene therapy in human patients.

### 3.5. Afatinib Inhibits Cell Growth and Enhances Apoptosis on SW579 Cells

**Figure 5:**
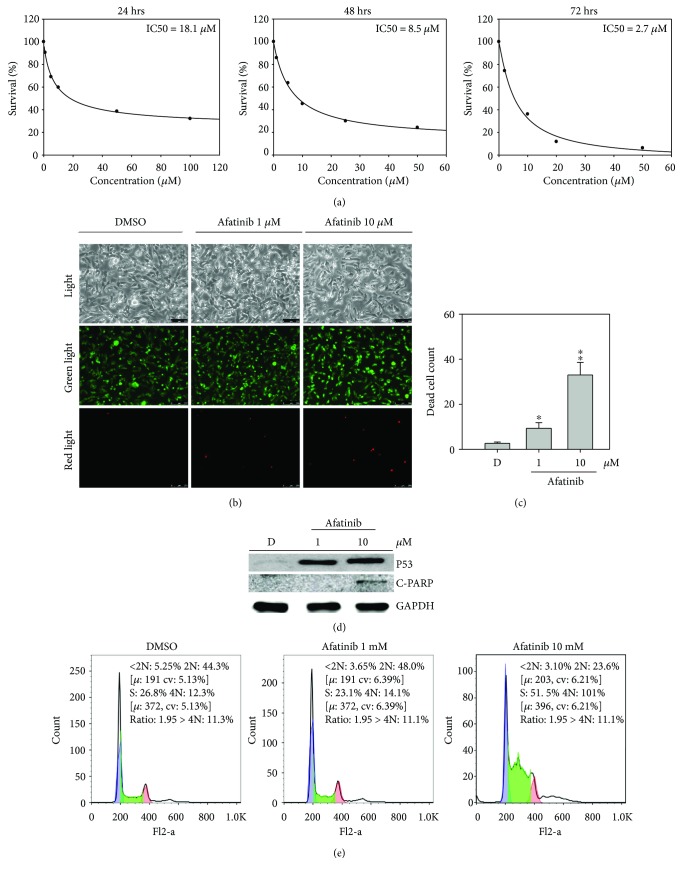
Afatinib inhibits SW579 cell survival and induces apoptosis. (a) The image shows the time-dependent manners of IC50 in afatinib-treated MDA-MB-231 cells by MTT assay at 24, 48, and 72 hours. (b) The LIVE/DEAD cell viability assay was performed after 1 and 10 *μ*M afatinib treatment of MDA-MB-231 cells for 48 hours. Cells were subjected to the viability assay to identify live (green) and dead (red) cells. (c) Cell death after 1 and 10 *μ*M afatinib treatment was analyzed for significance. (d) Afatinib significantly induced tumor-suppressive protein of P53 and cleaved PARP protein expression in a dose-dependent manner, as evidenced by western blot analysis. (e) The image shows the cell cycle distribution of afatinib-treated SW579 cells by flow cytometry analysis. Data are presented as the mean and standard error. Data were analyzed with Student's *t*-test; all *P* values were two-sided. *P* values less than 0.05 are indicated with an asterisk; less than 0.01 is presented with two asterisks.

Afatinib (Giotrif) is an irreversible pan-EGFR family blocker [25], which inhibits the tyrosine kinase activity of these receptors, resulting in reduced auto- and transphosphorylation within their dimerization and inhibition of critical steps in the signal transduction of all EGFR family members. To investigate the antianaplastic cancer activity of afatinib and the essential role of EGFR in SW579 cells in vitro, we determined the IC50 value of afatinib in SW579 cells by treating them with different concentrations of afatinib by MTT assay (Figure 5(a)). The result showed the cell viability of SW579 treatment for 48 and 72 hours was extremely sensitive to afatinib treatment with IC50 of 8.5 and 2.7 *μ*M, respectively. Next, we used a LIVE/DEAD assay to visualize afatinib-induced apoptosis (Figure 5(b)). The images showed an increasing number of SW579 cells undergoing apoptosis with increasing concentrations of afatinib, with a significant increase of 9.5 and 38-folds at 1 and 10 *μ*M afatinib treatments, compared to DMSO (Figure 5(c)). In addition, afatinib inhibited cell growth of SW579 through P53 activation and upregulation, eventually resulting in PARP cleavage and apoptosis (Figure 5(d)). In addition, the cell cycle distribution on SW579 also illustrated that the S phase arrest is significantly increased in 1 *μ*M and 10 *μ*M afatinib-treated cells, with 23.1% and 51.5% cell population, respectively (Figure 5(e)). The above evidence indicates that EGFR plays an important role in promoting cell growth and preventing apoptosis, implying that EGFR would be a perfect target for ATC therapy. Last, to clarify whether afatinib would induce EGFR-independent cell death on ATC cells, we measured the cell viability of SC and EGFR sgRNA_2-infected SW579 cells with 10 *μ*M afatinib treatment for 24 and 48 hours. In Supplementary Figure 2, it is clearly shown that either 24 or 48 hours afatinib exposure caused significant impacts on the cell survival of SC-infected cells, rather than that of EGFR sgRNA_2-infected cells (59 ± 2.5% versus 88 ± 3.6% at 24 hours, 43 ± 3.1% versus 74 ± 4.6% at 48 hours, *P*≦0.01). This result implies that afatinib as a multikinase target agent could potentially induce EGFR-independent cell death on SW579 cell, but EGFR genomic editing still would be the major reason to cause cell death on ATC cells.

## 4. Discussion

EGFR gene amplification is a frequent phenomenon occurring in many tumors, such as lung cancers [26], gastric cancer [27, 28], and breast cancers [29, 30]. Furthermore, high polysomy or gene mutation has been reported to play a role in EGFR overexpression in non-small-cell lung cancers and in breast cancer [31–33]. However, a few study has found a significant overexpression of VEGFR2 and EGFR in metastasis thyroid cancer by using IHC and FISH of clinical cancer tissues [34], even though there was not a complete agreement between EGFR protein expression and gene copy number, but with a significant correlation (*P* = 0.01). This study suggested that therapies targeting these tyrosine kinase receptors could have a clinical impact on these cancers. In the present study, using a large-scale analysis platform, we demonstrated that EGFR is predominately expressed in thyroid cancer by introducing gene microarray database of all 1036 cancer cells and TCGA clinical tumor information from 8215 tumors. Both observations well-characterized thyroid cancers that indicate EGFR participates in the progression of an important proportion of cases.

CRISPR-Cas9 RNA-guided nucleases are derived from an adaptive immune system that evolved in bacteria to defend against invading plasmids and viruses. The only limitation of the CRISPR/Cas system derives from the necessity of a protospacer-adjacent motif (PAM) located immediately 3′ to the target site, whereas the PAM sequence is specific to the species of Cas9.

CRISPR-Cas9-based knockout strategies are increasingly used to analyze gene function. In 2014, Yin and colleagues first reported the pioneer study to correct a genetic disease of type I tyrosinemia in postnatal animals by in vivo CRISPR/Cas9-mediated genome editing [35]. The authors delivered vectors that encode for Cas9 protein and the specific sgRNA to target mutated DNA sequence through mouse tail vein injection. The result of the animal study showed the correction of the mutant fumaryl-acetoacetate hydrolase (FAH) genes and stabilization of the FAH protein; the type I tyrosinemia mice were furtherly found to have therapeutic effects of reducing hepatocellular toxicity and a rescue in weight loss of mice. In this manuscript, we combined lentiviral transfection with CRISPR/Cas9 techniques to deliver vectors that encode for Cas9 protein and the specific sgRNA to EGFR DNA sequence in SW579 cells. The use of lentivirus-based CRISPR/Cas9 is to ensure the high infection efficiency on target cells and can be possibly applied in vivo and in further clinical therapy.

In this study, we found EGFR is overexpressed in thyroid cancer rather than the other types of cancer from CCLE and TCGA datasets. Using optimized lentivirus transfection condition, we conducted CRISPR/Cas9 gene-editing strategy to disrupt EGFR gene sequence and eliminate EGFR protein expression in both MDA-MB-231 cell and SW579 cells, ensuring that the high and specific gene-edit efficiency from our CRISPR/Cas9 system can be easily applied on any human cancer cells. Several genomic crosscheck experiments were performed to confirm the gene-editing efficiency in SW579, such as Sanger DNA sequencing, Tracking of Indels by Decomposition (TIDE) analysis, RGEN-RFLP, and western blot. Most importantly, using EGFR as a therapeutic target in metastatic progression either by TKI or genomic editing would provide solutions for the patients who suffered from the high risk of distant metastasis in ATC. Notably, the three-step therapeutic strategy of biomarker identification, current target-drug availability, and gene therapy development in this study provides a new potential ATC therapeutic strategy and the possibility of increasing ATC patient's survival rate with translational medicine.

## 5. Conclusions

Taken together, our study demonstrated that EGFR target therapy by using TKI or genomic editing suppressed cell viability and invasion ability on ATC cells. Signaling transduction analysis further uncovered that AKT and ERK activations are dependent on EGFR protein expression during ATC development. This provides new evidence that the target therapy on EGFR may be an effective therapeutic approach for ATC treatment in clinic. This study also suggested that several oncoproteins may be also targetable by using CRISPR/CAS9 for current and future clinical needs, which may possibly increase their survival rate in the translational medicine.

## Abbreviations

ATC: Aplastic thyroid cancer
TKIs: Tyrosine kinase inhibitors
CRISPR/Cas9: Clustered regularly interspaced short palindromic repeats
RGEN-RFLP: RNA-guided engineered nuclease-restriction fragment length polymorphism
TIDE: Tracking of Indels by Decomposition.

## Supplementary Materials

Supplementary 1 Supplementary Table: list of all QPCR, sgRNAs, and DNA PCR primers and their sequences.

Supplementary 2 Supplementary Figure 1: flow cytometry analysis of GFP-positive SW579 cells. GFP-positive SW579 cells were analyzed by flow cytometry. More GFP-positive cells were detected (right shift) with increasing pLJM1-EGFP virus copy number input (fold of SW579 cells) after three days.

Supplementary 3 Supplementary Figure 2: afatinib potentially induced EGFR-independent cell death on SW579 cell. The cell viability of SC and EGFR sgRNA_2 infected SW579 cells with 10 *μ*M afatinib treatment for 24 and 48 hours. The cell viability was carried out by MTT assay, and the data are presented as the mean and standard error. Data were analyzed with Student's *t*-test; all *P* values were two-sided. *P* values less than 0.01 are indicated with two asterisks.
