# Supplementary material for: CRISPR/Cas9 Genome Editing of Epidermal Growth Factor Receptor Sufficiently Abolished Oncogenicity in Anaplastic Thyroid Cancer
Source: Dis Markers. 2018 Apr 12;2018:3835783. doi: 10.1155/2018/3835783 (PMC5925145; doi:10.1155/2018/3835783)
Supplement: Supplementary 1 — Supplementary Table: list of all QPCR, sgRNAs, and DNA PCR primers and their sequences. [file 3835783.f1.docx]

**Supplementary table**

List of all QPCR, sgRNAs and DNA PCR primers and their sequences

| Primers | | Sequence |
| --- | --- | --- |
| Q-PCR  Primers |  | |
| GUS | Forward | 5' - AAACAGCCCGTTTACTTGAG |
|  | Reverse | 5' - AGTGTTCCTGCTAGAATAGATG |
| SNAI | Forward | 5' - CCCAATCGGAAGCCTAAC |
|  | Reverse | 5' - AGGACAGAGTCCCAGATGA |
| VIMEMTIN | Forward | 5' - ATTGAGATTGCCACCTACAG |
|  | Reverse | 5' - CCGTCTTAATCAGAAGTGTCC |
| TWIST | Forward | 5' - CAGCTATGTGGCTCACG |
|  | Reverse | 5' - TTCTCCTTCTCTGGAAACAATG |
| N-cadherin | Forward | 5' - CCAACAATTTCAGAGAGTACAACTT |
|  | Reverse | 5' - GATGCCATCAATGTCATCCT |
| Lentivirus WPRE | Forward | 5' - TCATGCTATTGCTTCCCGTA |
|  | Reverse | 5' - CCAAGGAAAGGACGATGAT |
| CRISPR Oligos |  | |
| Scramble | Forward | 5' - CACCGCCATATCGGGGCGAGACATG |
|  | Reverse | 5' - AAACCATGTCTCGCCCCGATATGGC |
| EGFR 1 | Forward | 5' - CACCGGAATTCGCTCCACTGTGTTG |
|  | Reverse | 5' - AAACCAACACAGTGGAGCGAATTCC |
| EGFR 2 | Forward | 5' - CACCGCGATCTCCACATCCTGCCGG |
|  | Reverse | 5' - AAACCCGGCAGGATGTGGAGATCGC |
| EGFR DNA Primers |  | |
| EGFR  Exon 3 | Forward | 5' - ATGACTGCAATCGTCTACC |
|  | Reverse | 5' - TCTGCCATCTTCTCAGTAACTT |
| EGFR  Exon 9 | Forward | 5' - GGAAATAATGAGGAGAACGCA |
|  | Reverse | 5' - ATGTGTGAAGGAGTCACTGAAA |
| FKRP DNA  Primers | Forward | 5' - TACAGCGAAAGCAACCACT |
|  | Reverse | 5' - GCTCTCCTCCTGCCAAT |
| RFLP Primers |  |  |
| EGFR 1 | Forward | 5' – GCGGCCTCTAATACGACTCACTATAGGGGAATTCGCTCCACTGTGTTGGTTTTAGAG |
| EGFR 2 | Forward | 5' - GCGGCCTCTAATACGACTCACTATAGGGCGATC TCCACATCCTGCCGGGTTTTAGAG |
| Universal | Reverse | 5' - AAAAAAGCACCGACTCG |
