# Supplementary figures and images for: CRISPR/Cas9 Genome Editing of Epidermal Growth Factor Receptor Sufficiently Abolished Oncogenicity in Anaplastic Thyroid Cancer
Source: Dis Markers. 2018 Apr 12;2018:3835783. doi: 10.1155/2018/3835783 (PMC5925145; doi:10.1155/2018/3835783)

Supplementary figure 1

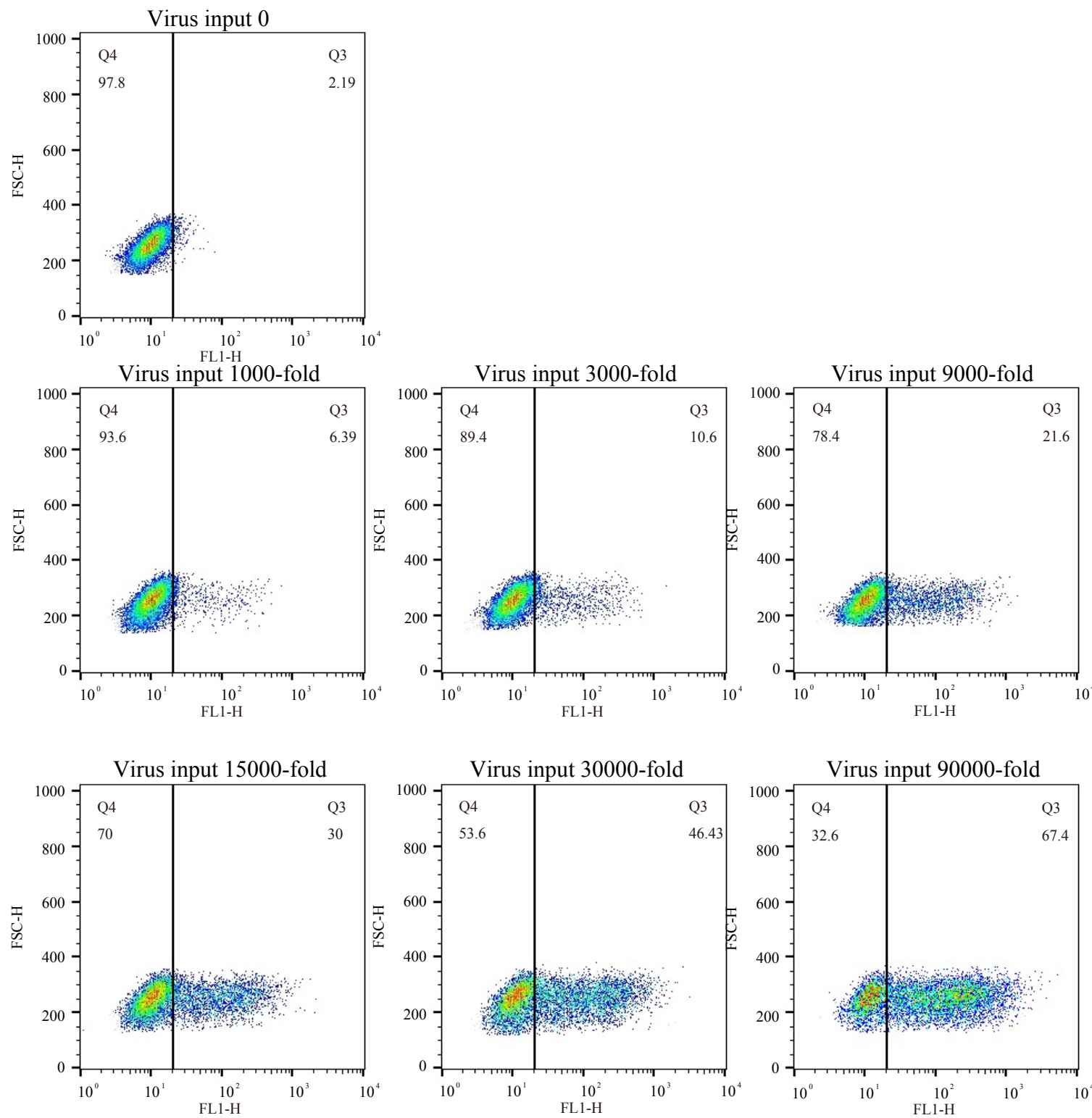

Supplement: Supplementary 2 — Supplementary Figure 1: flow cytometry analysis of GFP-positive SW579 cells. GFP-positive SW579 cells were analyzed by flow cytometry. More GFP-positive cells were detected (right shift) with increasing pLJM1-EGFP virus copy number input (fold of SW579 cells) after three days. [file 3835783.f2.pdf]

Supplementary figure 2

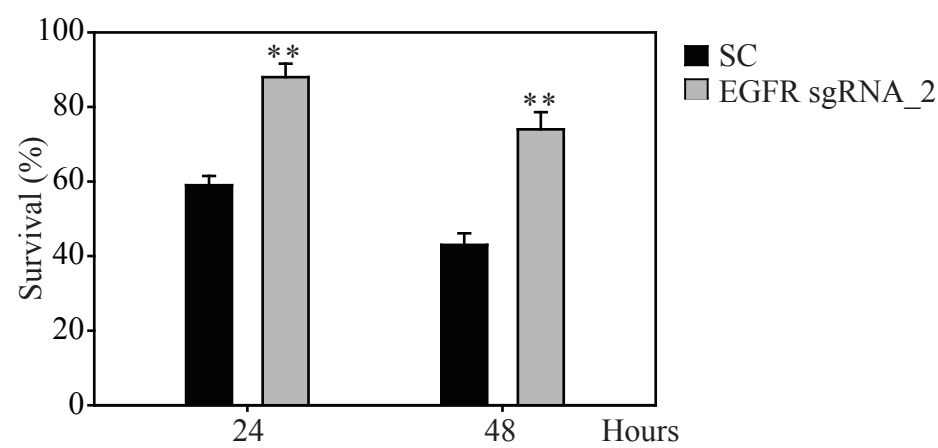

Supplement: Supplementary 3 — Supplementary Figure 2: afatinib potentially induced EGFR-independent cell death on SW579 cell. The cell viability of SC and EGFR sgRNA_2 infected SW579 cells with 10 μM afatinib treatment for 24 and 48 hours. The cell viability was carried out by MTT assay, and the data are presented as the mean and standard error. Data were analyzed with Student's t-test; all P values were two-sided. P values less than 0.01 are indicated with two asterisks. [file 3835783.f3.pdf]
